# Precision Oncology in Melanoma: Changing Practices

**DOI:** 10.2967/jnumed.124.267781

**Published:** 2024-12

**Authors:** Sean C. Dougherty, William L. Flowers, Elizabeth M. Gaughan

**Affiliations:** 1 Division of Hematology/Oncology, Department of Medicine, University of Virginia Health System, Charlottesville, Virginia; and; 2 Department of Radiology and Medical Imaging, University of Virginia Health System, Charlottesville, Virginia

**Keywords:** oncology, radioimmunoimaging, focused ultrasound, immunotherapy, melanoma staging, targeted therapy

## Abstract

Over the last 2 decades, significant progress has been made in our understanding of the genomics, tumor immune microenvironment, and immunogenicity of malignant melanoma. Historically, the prognosis for metastatic melanoma was poor because of limited treatment options. However, after multiple landmark clinical trials displaying the efficacy of combined *BRAF/MEK* inhibition for *BRAF*-mutant melanoma and the application of immune checkpoint inhibitors targeting the programmed death-1, cytotoxic T-lymphocyte antigen-4, and lymphocyte activation gene-3 molecules, overall survival rates have dramatically improved. The role of immune checkpoint inhibition has since expanded to the neoadjuvant and adjuvant settings with multiple regimens in routine use. Personalized therapies, including tumor-infiltrating lymphocytes that are extracted from a patient’s melanoma and eventually reinfused into the patient, and messenger RNA vaccines used to target neoantigens unique to a patient’s tumor, show promise. Improvements in accompanying imaging modalities, particularly within the field of nuclear medicine, have allowed for more accurate staging of disease and assessment of treatment response. Continued growth in the role of nuclear medicine in the evaluation of melanoma, including the incorporation of artificial intelligence into image interpretation and use of radiolabeled tracers allowing for intricate imaging of the tumor immune microenvironment, is expected in the coming years.

Malignant melanoma (hereafter melanoma) is an aggressive malignancy of pigment-producing cells called melanocytes (1). Melanocytes are found throughout the body in the skin, eyes, ears, gastrointestinal tract, and multiple mucous membranes, and thus 9 distinct subtypes of melanoma are recognized (2). Cutaneous melanoma, arising from melanocytes of the skin, accounts for the vast majority of melanoma diagnoses globally across all populations and ethnicities (3). Approximately 5% of all cases of melanoma are noncutaneous in origin; of these cases, 50% affect regions within the head and neck, with the nasal cavities and sinuses the main sites of disease (2). Uveal melanoma, the most common primary malignancy of the eye in adults, results from malignant transformation of melanocytes within the uveal tract, though it is pathophysiologically distinct from cutaneous melanoma (4). For the purposes of this review article, we are referring to cutaneous melanoma when addressing melanoma from this point onward.

The role of radiology in many areas of medicine, particularly oncology, continues to grow at a rapid pace. CT, ultrasound, and MRI play critical roles in the initial staging, treatment response assessment, and surveillance of many malignancies. The combination of ^18^F-FDG PET with low-dose CT, termed PET/CT, became commercially available in 2000 (5). PET/CT has resulted in significant improvements in treatment response assessment, correlation of areas of ^18^F-FDG uptake with underlying anatomic structures, and changes in surgical management in patients with resectable melanoma (6*,*^7^). Similarly, use of lymphoscintigraphy-assisted sentinel lymph node (LN) biopsy, initially developed as a minimally invasive technique for nodal staging in patients with melanoma, has been expanded to many solid tumor types (8*,*^9^).

## EPIDEMIOLOGY

Worldwide, there were 324,635 new cases of melanoma and 57,043 deaths from melanoma in 2020 (3). In the United States, age-adjusted rates of new cases of melanoma have been rising by 1.2% on average each year from 2010 to 2019. Despite this, because of significant improvements in earlier detection of disease and treatment options, death rates have been falling by 3.3% on average each year from 2011 to 2020 (10).

Notable differences in incidence, stage at time of diagnosis, and overall survival (OS) have been observed among non-Hispanic White and Black patients within the United States. Melanoma is more common among White patients than Black patients, with incidence rates of 30.6 and 0.9 cases per 100,00 individuals, respectively, from 2016 to 2020 (10). During this period, Black patients were more likely to be diagnosed with advanced disease, with 16.9% of cases with evidence of metastases at the time of diagnosis, compared with 4.8% of cases in White patients. Differences in OS exist between these 2 patients populations as well; when stratified by race and stage, Black patients had significantly lower OS for stage I and stage III disease than White patients (11).

Multiple risk factors for the development of melanoma have been established; exposure to ultraviolet radiation through sunlight and resultant sunburns is the most common. Ultraviolet radiation exposure through both ultraviolet A and ultraviolet B light results in DNA double-strand breaks and generation of reactive oxygen species that can indirectly damage DNA (12). Other risk factors include a family history of melanoma, certain phenotypic characteristics, and use of indoor tanning beds (13*,*^14^).

## STAGING OF DISEASE AT TIME OF DIAGNOSIS

Initial staging of biopsy-proven melanoma is performed according to the American Joint Committee on Cancer eighth edition staging system; this system retains the traditionally used characterizations of the primary tumor, regional LNs, and sites of distant metastases (15). Primary tumor size is reported as tumor (Breslow) thickness in millimeters. The presence or absence of ulceration, defined as the lack of intact epithelium over the melanoma, is prognostically relevant, as patient outcomes with ulcerated primary tumors are worse (16). Regional LNs are a common site of locoregional involvement of melanoma, and assessment for nodal disease is necessary for complete staging (17). The role of sentinel LN biopsy (SLNB) is discussed further later in the article.

Multiple imaging modalities are currently available for the staging of melanoma and evaluation for distant metastatic disease. Commonly used cross-sectional imaging techniques include contrast-enhanced CT of the neck, chest, abdomen, and pelvis; whole-body PET/CT; and MRI. Not all patients require radiographic evaluation for systemic disease; neither the National Comprehensive Cancer Network nor the American Academy of Dermatology recommends cross-sectional imaging for asymptomatic patients with stage 0–IIA melanoma (18*,*^19^). ^18^F-FDG PET has been shown insensitive for detection of occult LN metastases in these patient populations, with a sensitivity of only 13%–21% and a false-negative rate of 79%. The low sensitivity of ^18^F-FDG PET in this context is thought to be due to the small size of metastatic deposits in sentinel LNs, as the sensitivity improves with increases in lesion size and is near 100% for metastases larger than 10 mm (20*,*^21^).

**FIGURE 1.**
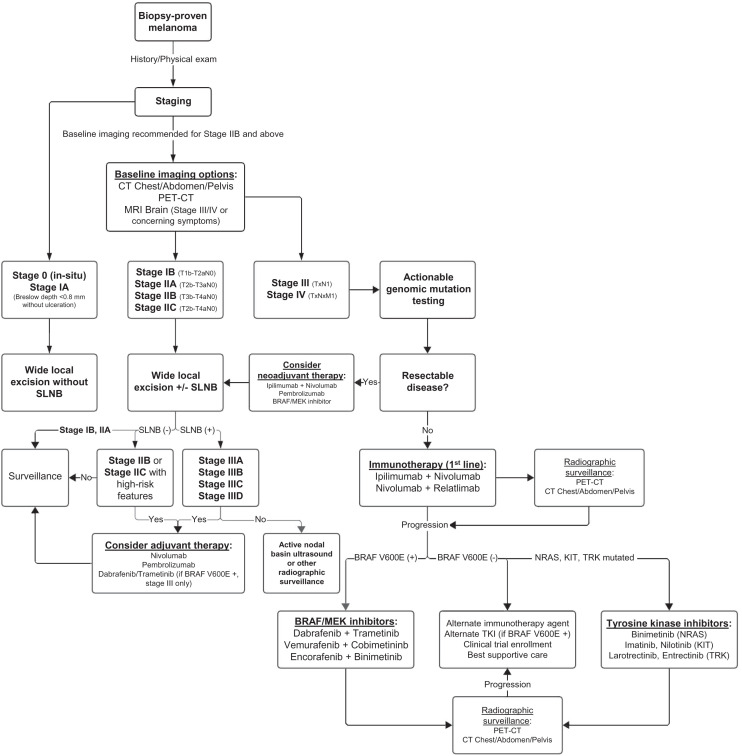
Treatment algorithm for melanoma from time of diagnosis and for multiple stages of disease.

Cross-sectional imaging for staging is recommended in patients with stage IIB disease or higher (Fig. 1) (18). Although a multitude of imaging techniques is available, robust data clearly delineating which modality should be used in specific clinical contexts are lacking, and clinical practice is heterogeneous (22). Despite a lack of randomized clinical trial data, however, ^18^F-FDG PET/CT has outperformed conventional imaging with contrast-enhanced CT for both initial staging and detection of recurrent disease in systematic reviews and metaanalyses (23*,*^24^). Comparisons to whole-body MRI have been mostly equivocal, with the current high costs and limited availability of whole-body MRI generally making it a less viable alternative (23). The exception is in cerebral metastasis, for which, because of the background high uptake of glucose, PET/CT is insensitive. For this reason, MRI of the brain is recommended for patients with stage III and IV disease and in all patients with symptoms concerning for intracranial metastases (18).

## MANAGEMENT OF PRIMARY CUTANEOUS MELANOMA

After a histologic diagnosis of melanoma and subsequent clinical staging, surgical resection via wide local excision remains the mainstay of treatment for early-stage melanoma (25). Wide excision is performed down to, but not including, the muscle fascia, and the recommended width of resected margins is dependent on the Breslow thickness (19). Surgical staging of regional LNs is a critical component of prognosis and systemic treatment planning for patients with localized melanoma, although it is not required in all patients. Current guidelines recommend lymphatic mapping and SLNB for all patients with a Breslow thickness greater than 1 mm (18). SLNB can also be considered for patients with a Breslow thickness between 0.8 and 1.0 mm or less than 0.8 mm when other high-risk features are present (18*,*^26^).

For patients undergoing SLNB, preoperative lymphatic mapping via lymphoscintigraphy allows for accurate identification of sentinel LNs for dissection and sampling. Initially pioneered by Morton et al., lymphoscintigraphy uses intradermal injections of dye and radiotracer at the primary tumor site, later allowing for intraoperative identification of sentinel LNs visually and by γ-probes (8). Historically, melanoma patients with a positive SLNB underwent complete LN dissection, which was associated with increased risk of lymphedema and decreased quality of life (27*,*^28^). Complete LN dissection versus observation was subsequently examined in 2 major multicenter clinical trials. The MSLT-NRASII and DeCOG-SLT studies showed that immediate complete LN dissection did not improve distant metastasis-free survival, relapse-free survival (RFS), or melanoma-specific survival compared with surveillance with delayed complete LN dissection at recurrence (29*,*^30^). Surveillance included ultrasonographic examination of the sentinel LN basin every 4 mo during the first 2 y and every 6 mo during years 3 through 5. The role of adjuvant therapy for high-risk patients with positive SLNB and other clinical features is discussed later in this article.

## MOLECULAR PROFILING AND TARGETED THERAPIES

**FIGURE 2.**
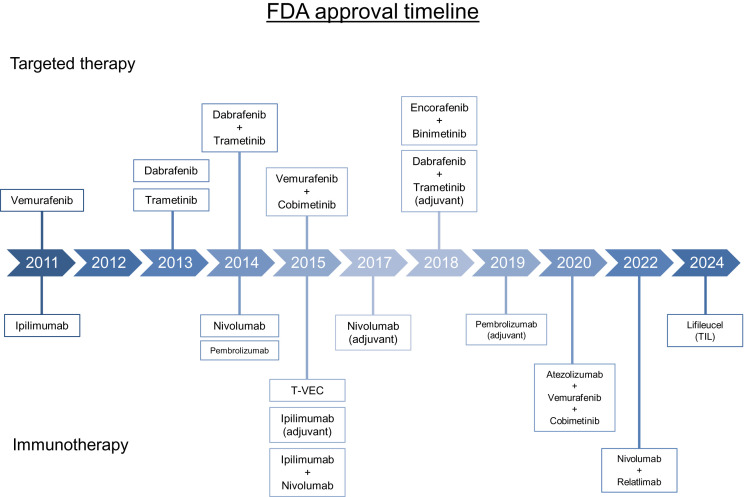
Timeline displaying FDA-approved targeted therapies and immunotherapies for melanoma. T-VEC = talimogene laherparepvec.

Molecular evaluation for the presence of driver mutations is the standard of care for most patients receiving systemic therapy in any setting. The most commonly observed mutation occurs in the *BRAF* oncogene; since its discovery in 2002, multiple targeted therapies for the treatment of melanoma have been approved (Fig. 2) (31). BRAF is a serine-threonine kinase within the RAS-RAF-MEK-ERK pathway; approximately 50% of melanomas harbor *BRAF V600* mutations resulting in constitutive activation of MEK and ERK, with *BRAF V600E* being the most commonly observed mutation (32). Other genomic mutations that have been identified include alterations in *NRAS* (∼28% of cases), *NF1* (∼14% of cases), and *KIT* (∼15%–20% of cases of acral or mucosal melanoma) (32*,*^33^). Molecular testing can be performed on tissue obtained from the primary tumor sample, tumor-involved regional LNs, or distant metastases.

Before the Food and Drug Administration (FDA) approval of the *BRAF* inhibitor vemurafenib in 2011, limited treatment options were available for metastatic melanoma, and mortality rates were high. The efficacy of oral *BRAF* inhibition was first shown by Flaherty et al. when multiple patients with *BRAF V600E*-mutated metastatic melanoma had complete or partial tumor regression with a *BRAF* inhibitor (34). The role of *MEK* inhibition in *BRAF*-mutant melanoma was then established when Flaherty et al. showed that trametinib, an oral *MEK* inhibitor, significantly improved progression-free survival (PFS) and OS compared with chemotherapy (35). Combination *BRAF* and *MEK* inhibition with dabrafenib plus trametinib was later shown to improve PFS and OS with a reduced toxicity profile and compared with dabrafenib alone (36). Multiple regimens using combination *BRAF* and *MEK* inhibition have been approved in the interim, and they remain important options for *BRAF*-mutant metastatic melanoma.

After the observed success of *BRAF* and *MEK* inhibition in the metastatic setting, the role of targeted therapy was examined in the adjuvant setting. In the COMBI-AD phase III trial, 870 patients with completely resected, stage III *BRAF*-mutated melanoma were randomized to receive oral dabrafenib plus trametinib or placebo for 12 mo after surgery. The 5-y analysis showed that RFS and distant metastasis-free survival were both longer in patients receiving dabrafenib plus trametinib, leading to FDA approval in 2018 (37). Patients with *BRAF*-mutated melanoma are also eligible for adjuvant immunotherapy; the optimal adjuvant therapy has not yet been established, as combination *BRAF* and *MEK* inhibition has not been directly compared with immunotherapy. The use of neoadjuvant *BRAF* plus *MEK* inhibition remains an ongoing area of investigation.

Therapies targeting mutations other than *BRAF* have been shown to be efficacious for metastatic melanoma in the second-line setting (Table 1). Approximately 15%–20% of cases of acral and mucosal melanoma will harbor an activating mutation in *KIT*. The tyrosine kinase inhibitor imatinib has been shown to increase PFS, with an overall disease control rate of approximately 55% when studied in small clinical trials and remains an option for patients who progress while on or are ineligible for immunotherapy and harbor a *KIT* mutation (38). Rarely, patients with cutaneous melanoma may harbor *TRK-*gene fusions and can receive subsequent-line therapy with *TRK*-gene fusion inhibitors such as larotrectinib and entrectinib (39*,*^40^). Mutations in *NRAS,* an oncogene found in the MAP kinase pathway, are found in approximately 15%–20% of *BRAF* wild-type cutaneous melanoma. Patients harboring *NRAS*-mutant tumors should receive front-line immunotherapy; however, if they have evidence of disease progression, off-label use of the *MEK* inhibitor binimetinib can be considered in addition to clinical trial enrollment (41).

**TABLE 1.** Actionable Genomic Mutations in Melanoma

Gene mutation	Tyrosine kinase inhibitor	Clinical trial	Outcome
BRAF/MEK	Dabrafenib + trametinib	COMBI-d (75)	3-y OS: 44% vs. 32% (dabrafenib + placebo) (HR, 0.75)
	Encorafenib + binimetinib	COLUMBUS (76)	5-y OS: 34.7% vs. 21.4% (vemurafenib)
	Vemurafenib + cobimetinib	coBRIM (77)	mOS: 22.3 vs. 17.4 mo (vemurafenib) (HR, 0.7)
NRAS	Binimetinib	NEMO (41)	mPFS: 2.8 vs. 1.5 mo (dacarbazine) (HR, 0.62)
KIT	Imatinib	Guo et al. (38)	mPFS: 3.5 mo with 1-y OS rate of 51%
	Nilotinib	Guo et al. (78)	mPFS: 4.2 mo and mOS: 18.0 mo
TRK	Larotrectinib	Hong et al. (39)	ORR: 79% of TRK-fusion–positive cancers
	Entrectinib	Doebele et al. (40)	ORR: 57% of TRK-fusion–positive cancers

HR = hazard ratio; mOS = median OS; mPFS = median PFS; ORR = overall response rate.

## IMMUNOTHERAPY AND IMMUNE CHECKPOINT INHIBITION

Advances in cancer immunotherapy have revolutionized the management of multiple malignancies, including melanoma. Studies of various forms of immunotherapy have been occurring for decades; however, it is only the recent progress in knowledge of cancer immunology and the tumor immune microenvironment that has propelled paradigm-changing treatments forward (42). Immune checkpoint inhibitors (ICIs) targeting the cytotoxic T-lymphocyte antigen-4 (CTLA-4), programmed death-1 (PD-1), and lymphocyte activation gene-3 (LAG-3) molecules are among the most prominent examples of such headway.

CTLA-4 and PD-1 are 2 key receptors expressed on T cells involved in regulating T-cell activity. CTLA-4 affects T cells during the initial stages of activation via its interaction with antigen-presenting cells. CTLA-4 competes with the T-cell costimulatory receptor cluster differentiation 28 for the binding of cluster differentiation 80 and 86, in turn decreasing T-cell activation (43). Shortly after their elucidation of the role of CTLA-4 in 1995, Allison et al. showed that blockade of the CTLA-4 molecule in mouse models resulted in enhanced antitumor immunity, spurring on multiple ensuing clinical trials in the years that would follow (44*,*^45^). PD-1 is an inhibitory receptor upregulated on activated T cells after long-term antigen exposure as part of homeostatic immune cell regulation (46). The binding of the programmed death ligand-1 (PD-L1) and programmed death ligand-2 molecules to the PD-1 receptor, which occurs primarily in chronically inflamed tissues, acts as a checkpoint for the adaptive immune system (47*,*^48^). However, tumor cells also exploit the PD-1/PD-L1 axis as a means of immune evasion. Recently, LAG-3 has emerged as a third promising target for ICIs. LAG-3 is expressed predominantly on exhausted T cells and negatively regulates T-cell activation and function. A multitude of randomized, placebo-controlled clinical trials examining the use of ICIs for the treatment of melanoma in the metastatic, adjuvant, and neoadjuvant settings has been performed after the discoveries of CTLA-4 and PD-1 by Allison and Nishimura (44*,*^46^).

## IMMUNE CHECKPOINT INHIBITION IN METASTATIC MELANOMA

The FDA approved the anti–CTLA-4 monoclonal antibody ipilimumab for the treatment of metastatic melanoma in 2011 after significant improvements in OS in multiple landmark trials (Table 2) (49*,*^50^). The role of combination immunotherapy in the metastatic setting was then examined in the CheckMate 067 trial when Wolchok et al. showed that the combination of ipilimumab plus nivolumab, an anti–PD-1 monoclonal antibody, was both safe and effective (51). Data later published showed that OS was significantly increased in patients receiving ipilimumab plus nivolumab compared with ipilimumab alone (52).

**TABLE 2.** ICIs for Treatment of Melanoma

ICI	Target	Landmark clinical trials	Typical use settings
Ipilimumab	CTLA-4	Hodi et al. (49); EORTC 18071 (54)	In combination with other agents as below
Nivolumab	PD-1	CheckMate 066 (79); CheckMate 238 (57)	Adjuvant, in combination with other agents
Pembrolizumab	PD-1	SWOG 1801 (63); KEYNOTE-054 (55)	Neoadjuvant, adjuvant
Ipilimumab + nivolumab	PD-1/CTLA-4	CheckMate 067 (51); NADINA (80)	Neoadjuvant, adjuvant, metastatic
Nivolumab + relatlimab	PD-1/LAG-3	RELATIVITY-047 (53)	Metastatic

The role of LAG-3 inhibition as a component of combination immunotherapy for metastatic melanoma was recently established in the RELATIVITY-047 trial (53). In patients with unresectable, stage III or IV melanoma, participants were randomly assigned to receive either relatlimab plus nivolumab or nivolumab monotherapy, both administered every 4 wk. The median PFS was 10.1 mo in the relatlimab-plus-nivolumab group compared with 4.6 mo in the nivolumab monotherapy group. OS data remain immature at this time.

## ADJUVANT IMMUNE CHECKPOINT INHIBITION

The role for ICIs in the adjuvant setting was first assessed by Eggermont et al. in the EORTC 18071 trial (54). In this phase III trial of patients with completely resected, stage III disease at high risk for recurrence, administration of ipilimumab every 3 wk for 4 doses and then every 3 mo for up to 3 y after surgery resulted in significantly longer RFS, with a median RFS of 26.1 mo in the ipilimumab group versus 17.1 mo in the placebo group (hazard ratio of 0.75). Results from this trial resulted in FDA approval of ipilimumab in the adjuvant setting in 2015.

The role of PD-1 blockade in the adjuvant setting was examined in the EORTC 1325/KEYNOTE-054 trial (55). In patients with completely resected stage III melanoma, pembrolizumab, an anti-PD-1 monoclonal antibody, administered every 3 wk for a total of 18 doses, was compared with placebo administered in a similar fashion. The 18-mo RFS was 71.4% in the pembrolizumab group versus 53.2% in the placebo group; in subgroup analysis, the risk of recurrence or death was shown to be 46% lower in patients with PD-L1–positive tumors treated with pembrolizumab than in those treated with placebo. Data from updated analyses published in 2021 confirmed improvement in RFS and distant metastasis-free survival with pembrolizumab, though OS data have not yet been reported (56).

The CheckMate 238 trial compared nivolumab with ipilimumab in a head-to-head fashion; 906 patients with completely resected stage III or IV melanoma were randomized to receive either adjuvant nivolumab or ipilimumab (57). Therapy was administered for up to 1 y or until recurrence of disease or unacceptable treatment-related toxicity. Initially, results favored nivolumab, as administration resulted in significantly longer RFS and distant metastasis-free survival than for ipilimumab. However, in an updated analysis published 4 y later, there was no significant difference in OS between the 2 cohorts, although RFS remained higher in the nivolumab arm (51.7% vs. 42.5%, hazard ratio of 0.71) (58).

After significant improvements in PFS and OS with use of combination immunotherapy in the metastatic setting (59), the role of ipilimumab plus nivolumab in the adjuvant space was studied in the CheckMate 915 trial. Patients with completely resected, stage III or IV disease were randomized to receive either ipilimumab plus nivolumab or nivolumab alone for up to 1 y (60). Ipilimumab plus nivolumab did not result in an improvement in RFS compared with nivolumab monotherapy (64.6% vs. 63.2%, hazard ratio of 0.92). Furthermore, rates of grade 3 or 4 treatment-related adverse events were higher in the combination arm than in the nivolumab monotherapy arm (32.6% vs. 12.8%). The role of combination immunotherapy incorporating a LAG-3 inhibitor is currently being studied in the RELATIVITY-098 trial, in which the anti-LAG-3 agent relatlimab plus nivolumab is being compared with nivolumab monotherapy (NCT05002569).

The role of personalized medicine in adjuvant immunotherapy for melanoma is expanding. In KEYNOTE-942, Weber et al. recently showed that creation and administration of an individualized messenger RNA vaccine plus pembrolizumab improved RFS compared with pembrolizumab alone (61). The messenger RNA-4157 vaccine, generated by harvesting patient tumor samples, performing whole-exome sequencing, and identifying tumor-specific mutations, encodes up to 34 neoantigens in a lipid nanoparticle formulation. Similarly, administration of autologous tumor-infiltrating lymphocytes, initially extracted from a patient’s melanoma and then expanded ex vivo, was recently FDA-approved for metastatic melanoma previously treated with at least one line of systemic therapy (62).

## NEOADJUVANT IMMUNE CHECKPOINT INHIBITION

Recently, practice-changing clinical trials exploring the role of neoadjuvant immunotherapy have demonstrated the benefit of ICIs in this setting. In the landmark phase II SWOG S1801 trial, 313 patients with resectable stage IIIB–IV melanoma with clinically detected or radiographically evident LN involvement were randomized to receive pembrolizumab, administered every 3 wk for a total of 3 doses before surgery, followed by 15 doses of pembrolizumab as adjuvant therapy or surgical resection followed by adjuvant pembrolizumab every 3 wk for a total of 18 doses (63). At the median follow-up of 15 mo, patients who received neoadjuvant pembrolizumab had improved EFS compared with those who received adjuvant therapy alone, with 2-y EFS of 72% versus 49%, respectively, with a hazard ratio of 0.58. The benefit of neoadjuvant pembrolizumab was seen across all subgroups, including patients with *BRAF*-mutated and wild-type disease, and treatment-related toxicities resulting in an inability to undergo surgery occurred in less than 10% of patients. OS data from this trial remain immature at this time.

## IMAGING SURVEILLANCE OF CUTANEOUS MELANOMA

Given the historical lack of effective treatments for melanoma, there has been a paucity of randomized clinical trials conducted on imaging surveillance. As such, little evidence currently exists regarding optimal modality or timeline. As a result, the role of imaging surveillance in melanoma lacks expert agreement, reflected in national and international guideline heterogeneity. Consistent among guidelines, however, is the recommendation against surveillance imaging in early-stage disease, for which imaging detection rates have been shown to be lower than false-positive rates (64). Most guidelines do recommend surveillance imaging with stage IIB or IIC disease, for which rates of distant recurrence are significantly increased (18).

Regarding preferred imaging modality, evidence for the improved accuracy of PET/CT compared with conventional contrast-enhanced CT in melanoma surveillance is strong. A 2011 systematic review demonstrated that the sensitivity and specificity of PET/CT was 86% and 91%, compared with 63% and 78% for CT of the chest, abdomen, and pelvis (65). The continued recommendation for CT of the chest, abdomen, and pelvis by some guidelines has been explained as a reflection of the limited availability of PET/CT in certain regions and demographics. Contrast-enhanced CT, and potentially whole-body MRI, can be considered in this context.

**FIGURE 3.**
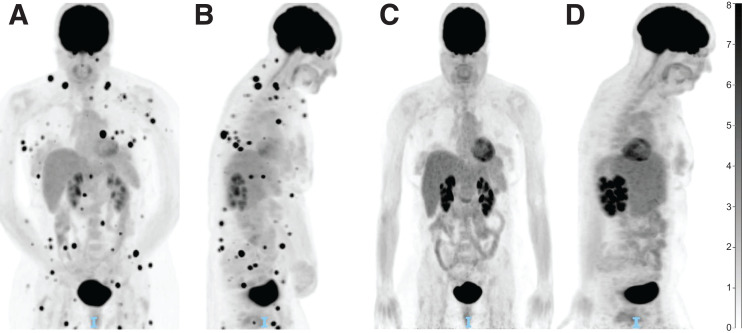
PET/CT for immunotherapy treatment response assessment. (A) PET/CT displaying innumerable intramuscular and subcutaneous soft-tissue nodules consistent with metastatic melanoma (coronal view). (B) Initial PET/CT (midsagittal view). (C) Repeat PET/CT less than 3 mo later and after 3 cycles of ipilimumab-nivolumab, showing complete resolution of soft-tissue nodules (coronal view). (D) Repeat PET/CT displaying treatment response (midsagittal view). Unit of measure for intensity bar is SUV = C(T)/[injection dose (MBq)/patient weight (kg)]).

In patients treated with ICI, ^18^F-FDG PET appears to be particularly useful in the monitoring and prediction of response. Using RECIST, the most validated CT criteria in use for solid tumors, multiple ^18^F-FDG PET criteria have been adapted for the interpretation of immunotherapy response, most recently with PET Response Evaluation Criteria for Immunotherapy and immune PERCIST. Though validation is ongoing, all of these criteria ultimately conclude that metabolic response in ^18^F-FDG PET is strongly associated with survival outcomes (66). The recently published joint guidelines from the European Association of Nuclear Medicine, Society of Nuclear Medicine and Molecular Imaging, and Australian and New Zealand Society of Nuclear Medicine therefore endorse ^18^F-FDG PET/CT as the imaging modality of choice for baseline disease assessment before the start of immunotherapy. ^18^F-FDG PET/CT is also recommended after approximately 3–4 cycles of treatment (Fig. 3) in the case of clinical deterioration or suspected progression on other imaging, before restarting treatment in cases of temporary interruption, and before treatment discontinuation (67).

**FIGURE 4.**
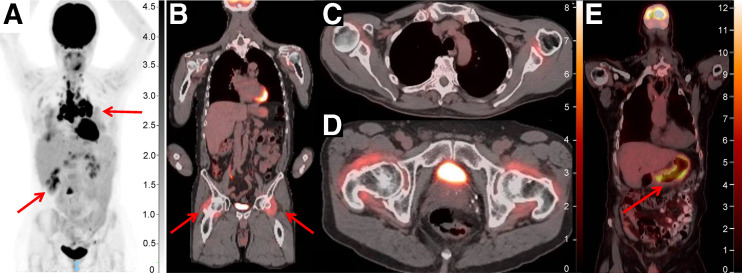
PET/CT for detection of immune-related adverse events. (A) PET/CT displaying hypermetabolic pulmonary nodules, subcarinal and hilar adenopathy, and hypermetabolic liver lesions (arrows), initially concerning for metastatic disease but later biopsied and found to be consistent with ICI-related sarcoidosis. (B) Increased ^18^F-FDG uptake bilaterally in hips (arrows) and shoulders consistent with immune-mediated arthritis. (C and D) Two axial views of ^18^F-FDG uptake in shoulders and hips. (E) Diffuse ^18^F-FDG uptake within posterior and posterior-lateral gastric wall consistent with immune-mediated gastritis, noted before onset of symptoms. Unit of measure for intensity bar is SUV = C(T)/[injection dose (MBq)/patient weight (kg)]).

Given the novel mechanisms of action of ICIs, atypical treatment responses not seen with use of traditional chemotherapy or other targeted therapies have been observed; these include pseudoprogression, hyperprogression, and immune-related adverse events. ^18^F-FDG PET/CT is again uniquely advantageous in this context given its ability to characterize metabolically active tissue. Pseudoprogression, defined as an initial transient increase in tumor metabolic activity and morphologic size followed by a true response, occurs in approximately 10% of patients treated with ICIs. It is seen mostly frequently within the first 4–6 wk after initiation of treatment but can occur up to several months thereafter (67). Differentiating pseudoprogression from true progression requires follow-up imaging 4–8 wk later. Conversely, hyperprogression—an atypical, true acceleration of tumor growth on imaging after initiation of immunotherapy—represents rapid treatment failure and has been observed in melanoma and other malignancies. Efforts to define and understand the mechanism of this phenomenon continue; however, it is thought to occur in approximately 4%–26% of cases when defined by doubling of volume or growth rate on imaging (68*,*^69^). Finally, ^18^F-FDG PET has also been shown to be uniquely effective in identifying immune-related adverse events, including detection before onset of symptoms (Fig. 4) (67).

## FUTURE OF RADIOLOGY IN MELANOMA

Recently, the use of high-intensity focused ultrasound as a noninvasive form of tumor ablation has been proposed as an adjunct therapy for multiple solid tumors, including melanoma. An ultrasound transducer positioned outside the body or within a body cavity is used to focus high-intensity ultrasound beams in a small region of tumor, resulting in extreme intratumoral temperature elevations in a matter of seconds, ultimately causing necrosis of tumor cells (70). In addition to a reduction in tumor burden, high-intensity focused ultrasound results in the release of tumor antigens and damage-associated molecular patterns, triggering both innate and adaptive immune responses. Studies evaluating optimization of high-intensity focused ultrasound protocols and incorporation of immunotherapy in addition to high-intensity focused ultrasound are ongoing (NCT04116320).

Current investigations into the role of artificial intelligence and machine learning in ^18^F-FDG PET/CT are promising. One area in which machine learning has proven particularly useful is in the identification of novel metabolic markers on ^18^F-FDG PET to guide treatment strategies. Multiple studies on one such parameter, total metabolic tumor volume, calculated by both manual and automated lesion segmentation, have demonstrated significant correlation with poor treatment response to pembrolizumab (71). Another marker with apparent significant prognostic value is high metabolic activity of hematopoietic tissues, such as bone marrow and spleen, which has been correlated with poor response to ICIs and an immunosuppressive environment in multiple studies (72).

The development of novel radiolabeled tracers to detect malignancy, guide therapy, and identify cellular microenvironments is ongoing. Numerous tracers are being studied in various preclinical and clinical stages of development. Melanin imaging, although suggested in recent studies to be a more complex target than initially thought, is highly specific to melanoma patients. The labeling of ^18^F to benzamide derivatives such as 5-FPN (^18^F-5-fluoro-*N*-[2-(diethylamino)ethyl]picolinamide) and MEL050 (^18^F-6-fluoro-*N*-[2-(diethylamino)ethyl]pyridine-3-carboxamide) has demonstrated in vivo imaging performance superior to ^18^F-FDG (73). Melanin-targeted therapy has also demonstrated early promise, with ^131^I and ^188^Re labeled to various molecules demonstrating antitumor efficacy with limited toxicities (73). Multiple tracers, including ^68^Ga, ^89^Zr, and ^18^F, have also been attached to monoclonal antibodies against PD-1 and PD-L1, allowing for a noninvasive whole-body map of immune checkpoint proteins. These radiolabeled tracers have the potential to be used to stratify treatment candidates, monitor therapy, and create a pathway for targeted therapy (74).

## References

[1] Lin JY Fisher DE. Melanocyte biology and skin pigmentation. Nature. 2007;445:843–850. 17314970 10.1038/nature05660

[2] Elder DE Bastian BC Cree IA Massi D Scolyer RA. The 2018 World Health Organization classification of cutaneous, mucosal, and uveal melanoma: detailed analysis of 9 distinct subtypes defined by their evolutionary pathway. Arch Pathol Lab Med. 2020;144:500–522. 32057276 10.5858/arpa.2019-0561-RA

[3] Sung H Ferlay J Siegel RL. Global cancer statistics 2020: GLOBOCAN estimates of incidence and mortality worldwide for 36 cancers in 185 countries. CA Cancer J Clin. 2021;71:209–249. 33538338 10.3322/caac.21660

[4] Carvajal RD Sacco JJ Jager MJ. Advances in the clinical management of uveal melanoma. Nat Rev Clin Oncol. 2023;20:99–115. 36600005 10.1038/s41571-022-00714-1

[5] Beyer T Townsend DW Brun T. A combined PET/CT scanner for clinical oncology. J Nucl Med. 2000;41:1369–1379. 10945530

[6] Perissinotti A Vidal-Sicart S Nieweg O Valdes Olmos R. Melanoma and nuclear medicine. Melanoma Manag. 2014;1:57–74. 30190811 10.2217/mmt.14.10 PMC6094667

[7] Alvarez Paez AM Brouwer OR Veenstra HJ. Decisive role of SPECT/CT in localization of unusual periscapular sentinel nodes in patients with posterior trunk melanoma: three illustrative cases and a review of the literature. Melanoma Res. 2012;22:278–283. 22456165 10.1097/CMR.0b013e32835312b1

[8] Morton DL Wen DR Wong JH. Technical details of intraoperative lymphatic mapping for early stage melanoma. Arch Surg. 1992;127:392–399. 1558490 10.1001/archsurg.1992.01420040034005

[9] Morton DL Hoon DS Cochran AJ. Lymphatic mapping and sentinel lymphadenectomy for early-stage melanoma: therapeutic utility and implications of nodal microanatomy and molecular staging for improving the accuracy of detection of nodal micrometastases. Ann Surg. 2003;238:538–549. 14530725 10.1097/01.sla.0000086543.45557.cb PMC1360112

[10] SEER*Explorer. National Cancer Institute website. https://seer.cancer.gov/statistics-network/explorer/application.html. Updated June 27, 2024. Accessed October 23, 2024.

[11] Dawes SM Tsai S Gittleman H Barnholtz-Sloan JS Bordeaux JS. Racial disparities in melanoma survival. J Am Acad Dermatol. 2016;75:983–991. 27476974 10.1016/j.jaad.2016.06.006

[12] Anna B Blazej Z Jacqueline G Andrew CJ Jeffrey R Andrzej S. Mechanism of UV-related carcinogenesis and its contribution to nevi/melanoma. Expert Rev Dermatol. 2007;2:451–469. 18846265 10.1586/17469872.2.4.451 PMC2564815

[13] Elwood JM Gallagher RP. Body site distribution of cutaneous malignant melanoma in relationship to patterns of sun exposure. Int J Cancer. 1998;78:276–280. 9766557 10.1002/(SICI)1097-0215(19981029)78:3<276::AID-IJC2>3.0.CO;2-S

[14] Gandini S Sera F Cattaruzza MS. Meta-analysis of risk factors for cutaneous melanoma: III. Family history, actinic damage and phenotypic factors. Eur J Cancer. 2005;41:2040–2059. 16125929 10.1016/j.ejca.2005.03.034

[15] Gershenwald JE Scolyer RA Hess KR. Melanoma staging: evidence-based changes in the American Joint Committee on Cancer eighth edition cancer staging manual. CA Cancer J Clin. 2017;67:472–492. 29028110 10.3322/caac.21409 PMC5978683

[16] Balch CM Wilkerson JA Murad TM Soong SJ Ingalls AL Maddox WA. The prognostic significance of ulceration of cutaneous melanoma. Cancer. 1980;45:3012–3017. 7388745 10.1002/1097-0142(19800615)45:12<3012::aid-cncr2820451223>3.0.co;2-o

[17] Meier F Will S Ellwanger U. Metastatic pathways and time courses in the orderly progression of cutaneous melanoma. Br J Dermatol. 2002;147:62–70. 12100186 10.1046/j.1365-2133.2002.04867.x

[18] Melanoma: cutaneous. NCCN website. https://www.nccn.org/professionals/physician_gls/pdf/cutaneous_melanoma.pdf. Published September 23, 2024. Accessed October 23, 2024.

[19] Swetter SM Tsao H Bichakjian CK. Guidelines of care for the management of primary cutaneous melanoma. J Am Acad Dermatol. 2019;80:208–250. 30392755 10.1016/j.jaad.2018.08.055

[20] Wagner JD Schauwecker D Davidson D. Inefficacy of F-18 fluorodeoxy-D-glucose-positron emission tomography scans for initial evaluation in early-stage cutaneous melanoma. Cancer. 2005;104:570–579. 15977211 10.1002/cncr.21189

[21] Crippa F Leutner M Belli F. Which kinds of lymph node metastases can FDG PET detect? A clinical study in melanoma. J Nucl Med. 2000;41:1491–1494. 10994727

[22] Dinnes J Ferrante di Ruffano L Takwoingi Y. Ultrasound, CT, MRI, or PET-CT for staging and re-staging of adults with cutaneous melanoma. Cochrane Database Syst Rev. 2019;7:CD012806. 31260100 10.1002/14651858.CD012806.pub2 PMC6601698

[23] Bisschop C de Heer EC Brouwers AH Hospers GAP Jalving M. Rational use of ^18^F-FDG PET/CT in patients with advanced cutaneous melanoma: a systematic review. Crit Rev Oncol Hematol. 2020;153:103044. 32673997 10.1016/j.critrevonc.2020.103044

[24] Perng P Marcus C Subramaniam RM. ^18^F-FDG PET/CT and melanoma: staging, immune modulation and mutation-targeted therapy assessment, and prognosis. AJR. 2015;205:259–270. 26204273 10.2214/AJR.14.13575

[25] Veronesi U Cascinelli N Adamus J. Thin stage I primary cutaneous malignant melanoma. Comparison of excision with margins of 1 or 3 cm. N Engl J Med. 1988;318:1159–1162. 3079582 10.1056/NEJM198805053181804

[26] Ross MI Gershenwald JE. Evidence-based treatment of early-stage melanoma. J Surg Oncol. 2011;104:341–353. 21858828 10.1002/jso.21962

[27] Morton DL Cochran AJ Thompson JF. Sentinel node biopsy for early-stage melanoma: accuracy and morbidity in MSLT-I, an international multicenter trial. Ann Surg. 2005;242:302–311. 16135917 10.1097/01.sla.0000181092.50141.fa PMC1357739

[28] Wrightson WR Wong SL Edwards MJ. Complications associated with sentinel lymph node biopsy for melanoma. Ann Surg Oncol. 2003;10:676–680. 12839853 10.1245/aso.2003.10.001

[29] Faries MB Thompson JF Cochran AJ. Completion dissection or observation for sentinel-node metastasis in melanoma. N Engl J Med. 2017;376:2211–2222. 28591523 10.1056/NEJMoa1613210 PMC5548388

[30] Leiter U Stadler R Mauch C. Final analysis of DeCOG-SLT trial: no survival benefit for complete lymph node dissection in patients with melanoma with positive sentinel node. J Clin Oncol. 2019;37:3000–3008. 31557067 10.1200/JCO.18.02306

[31] Davies H Bignell GR Cox C. Mutations of the BRAF gene in human cancer. Nature. 2002;417:949–954. 12068308 10.1038/nature00766

[32] Cancer Genome Atlas Network. Genomic classification of cutaneous melanoma. Cell. 2015;161:1681–1696. 26091043 10.1016/j.cell.2015.05.044 PMC4580370

[33] Curtin JA Busam K Pinkel D Bastian BC. Somatic activation of KIT in distinct subtypes of melanoma. J Clin Oncol. 2006;24:4340–4346. 16908931 10.1200/JCO.2006.06.2984

[34] Flaherty KT Puzanov I Kim KB. Inhibition of mutated, activated BRAF in metastatic melanoma. N Engl J Med. 2010;363:809–819. 20818844 10.1056/NEJMoa1002011 PMC3724529

[35] Flaherty KT Robert C Hersey P. Improved survival with MEK inhibition in BRAF-mutated melanoma. N Engl J Med. 2012;367:107–114. 22663011 10.1056/NEJMoa1203421

[36] Long GV Flaherty KT Stroyakovskiy D. Dabrafenib plus trametinib versus dabrafenib monotherapy in patients with metastatic BRAF V600E/K-mutant melanoma: long-term survival and safety analysis of a phase 3 study. Ann Oncol. 2019;30:1848. 31406976 10.1093/annonc/mdz221 PMC6927319

[37] Dummer R Hauschild A Santinami M. Five-year analysis of adjuvant dabrafenib plus trametinib in stage III melanoma. N Engl J Med. 2020;383:1139–1148. 32877599 10.1056/NEJMoa2005493

[38] Guo J Si L Kong Y. Phase II, open-label, single-arm trial of imatinib mesylate in patients with metastatic melanoma harboring c-Kit mutation or amplification. J Clin Oncol. 2011;29:2904–2909. 21690468 10.1200/JCO.2010.33.9275

[39] Hong DS DuBois SG Kummar S. Larotrectinib in patients with TRK fusion-positive solid tumours: a pooled analysis of three phase 1/2 clinical trials. Lancet Oncol. 2020;21:531–540. 32105622 10.1016/S1470-2045(19)30856-3 PMC7497841

[40] Doebele RC Drilon A Paz-Ares L. Entrectinib in patients with advanced or metastatic NTRK fusion-positive solid tumours: integrated analysis of three phase 1-2 trials. Lancet Oncol. 2020;21:271–282. 31838007 10.1016/S1470-2045(19)30691-6 PMC7461630

[41] Dummer R Schadendorf D Ascierto PA. Binimetinib versus dacarbazine in patients with advanced NRAS-mutant melanoma (NEMO): a multicentre, open-label, randomised, phase 3 trial. Lancet Oncol. 2017;18:435–445. 28284557 10.1016/S1470-2045(17)30180-8

[42] Dobosz P Dzieciatkowski T. The intriguing history of cancer immunotherapy. Front Immunol. 2019;10:2965. 31921205 10.3389/fimmu.2019.02965 PMC6928196

[43] Linsley PS Greene JL Tan P. Coexpression and functional cooperation of CTLA-4 and CD28 on activated T lymphocytes. J Exp Med. 1992;176:1595–1604. 1334116 10.1084/jem.176.6.1595 PMC2119471

[44] Krummel MF Allison JP. CD28 and CTLA-4 have opposing effects on the response of T cells to stimulation. J Exp Med. 1995;182:459–465. 7543139 10.1084/jem.182.2.459 PMC2192127

[45] Leach DR Krummel MF Allison JP. Enhancement of antitumor immunity by CTLA-4 blockade. Science. 1996;271:1734–1736. 8596936 10.1126/science.271.5256.1734

[46] Nishimura H Nose M Hiai H Minato N Honjo T. Development of lupus-like autoimmune diseases by disruption of the PD-1 gene encoding an ITIM motif-carrying immunoreceptor. Immunity. 1999;11:141–151. 10485649 10.1016/s1074-7613(00)80089-8

[47] Sharpe AH Pauken KE. The diverse functions of the PD1 inhibitory pathway. Nat Rev Immunol. 2018;18:153–167. 28990585 10.1038/nri.2017.108

[48] Iwai Y Ishida M Tanaka Y Okazaki T Honjo T Minato N. Involvement of PD-L1 on tumor cells in the escape from host immune system and tumor immunotherapy by PD-L1 blockade. Proc Natl Acad Sci USA. 2002;99:12293–12297. 12218188 10.1073/pnas.192461099 PMC129438

[49] Hodi FS O’Day SJ McDermott DF. Improved survival with ipilimumab in patients with metastatic melanoma. N Engl J Med. 2010;363:711–723. 20525992 10.1056/NEJMoa1003466 PMC3549297

[50] Robert C Thomas L Bondarenko I. Ipilimumab plus dacarbazine for previously untreated metastatic melanoma. N Engl J Med. 2011;364:2517–2526. 21639810 10.1056/NEJMoa1104621

[51] Wolchok JD Kluger H Callahan MK. Nivolumab plus ipilimumab in advanced melanoma. N Engl J Med. 2013;369:122–133. 23724867 10.1056/NEJMoa1302369 PMC5698004

[52] Wolchok JD Chiarion-Sileni V Gonzalez R. Overall survival with combined nivolumab and ipilimumab in advanced melanoma. N Engl J Med. 2017;377:1345–1356. 28889792 10.1056/NEJMoa1709684 PMC5706778

[53] Tawbi HA Schadendorf D Lipson EJ. Relatlimab and nivolumab versus nivolumab in untreated advanced melanoma. N Engl J Med. 2022;386:24–34. 34986285 10.1056/NEJMoa2109970 PMC9844513

[54] Eggermont AM Chiarion-Sileni V Grob JJ. Adjuvant ipilimumab versus placebo after complete resection of high-risk stage III melanoma (EORTC 18071): a randomised, double-blind, phase 3 trial. Lancet Oncol. 2015;16:522–530. 25840693 10.1016/S1470-2045(15)70122-1

[55] Eggermont AMM Blank CU Mandala M. Adjuvant pembrolizumab versus placebo in resected stage III melanoma. N Engl J Med. 2018;378:1789–1801. 29658430 10.1056/NEJMoa1802357

[56] Eggermont AMM Blank CU Mandala M. Adjuvant pembrolizumab versus placebo in resected stage III melanoma (EORTC 1325-MG/KEYNOTE-054): distant metastasis-free survival results from a double-blind, randomised, controlled, phase 3 trial. Lancet Oncol. 2021;22:643–654. 33857412 10.1016/S1470-2045(21)00065-6

[57] Weber J Mandala M Del Vecchio M. Adjuvant nivolumab versus ipilimumab in resected stage III or IV melanoma. N Engl J Med. 2017;377:1824–1835. 28891423 10.1056/NEJMoa1709030

[58] Ascierto PA Del Vecchio M Mandala M. Adjuvant nivolumab versus ipilimumab in resected stage IIIB-C and stage IV melanoma (CheckMate 238): 4-year results from a multicentre, double-blind, randomised, controlled, phase 3 trial. Lancet Oncol. 2020;21:1465–1477. 32961119 10.1016/S1470-2045(20)30494-0

[59] Larkin J Chiarion-Sileni V Gonzalez R. Five-year survival with combined nivolumab and ipilimumab in advanced melanoma. N Engl J Med. 2019;381:1535–1546. 31562797 10.1056/NEJMoa1910836

[60] Weber JS Schadendorf D Del Vecchio M. Adjuvant therapy of nivolumab combined with ipilimumab versus nivolumab alone in patients with resected stage IIIB-D or stage IV melanoma (CheckMate 915). J Clin Oncol. 2023;41:517–527. 36162037 10.1200/JCO.22.00533 PMC9870220

[61] Weber JS Carlino MS Khattak A. Individualised neoantigen therapy mRNA-4157 (V940) plus pembrolizumab versus pembrolizumab monotherapy in resected melanoma (KEYNOTE-942): a randomised, phase 2b study. Lancet. 2024;403:632–644. 10.1016/S0140-6736(23)02268-7 38246194

[62] Betof Warner A Corrie PG Hamid O. Tumor-infiltrating lymphocyte therapy in melanoma: facts to the future. Clin Cancer Res. 2023;29:1835–1854. 36485001 10.1158/1078-0432.CCR-22-1922 PMC10183807

[63] Patel SP Othus M Chen Y. Neoadjuvant-adjuvant or adjuvant-only pembrolizumab in advanced melanoma. N Engl J Med. 2023;388:813–823. 36856617 10.1056/NEJMoa2211437 PMC10410527

[64] Johnston L Starkey S Mukovozov I Robertson L Petrella T Alhusayen R. Surveillance after a previous cutaneous melanoma diagnosis: a scoping review of melanoma follow-up guidelines. J Cutan Med Surg. 2023;27:516–525. 37489919 10.1177/12034754231188434 PMC10617001

[65] Xing Y Bronstein Y Ross MI. Contemporary diagnostic imaging modalities for the staging and surveillance of melanoma patients: a meta-analysis. J Natl Cancer Inst. 2011;103:129–142. 21081714 10.1093/jnci/djq455 PMC3022618

[66] Sachpekidis C Weru V Kopp-Schneider A Hassel JC Dimitrakopoulou-Strauss A. The prognostic value of [^18^F]FDG PET/CT based response monitoring in metastatic melanoma patients undergoing immunotherapy: comparison of different metabolic criteria. Eur J Nucl Med Mol Imaging. 2023;50:2699–2714. 37099131 10.1007/s00259-023-06243-y PMC10317882

[67] Lopci E Hicks RJ Dimitrakopoulou-Strauss A. Joint EANM/SNMMI/ANZSNM practice guidelines/procedure standards on recommended use of [^18^F]FDG PET/CT imaging during immunomodulatory treatments in patients with solid tumors version 1.0. Eur J Nucl Med Mol Imaging. 2022;49:2323–2341. 35376991 10.1007/s00259-022-05780-2 PMC9165250

[68] Kato S Goodman A Walavalkar V Barkauskas DA Sharabi A Kurzrock R. Hyperprogressors after immunotherapy: analysis of genomic alterations associated with accelerated growth rate. Clin Cancer Res. 2017;23:4242–4250. 28351930 10.1158/1078-0432.CCR-16-3133 PMC5647162

[69] Saâda-Bouzid E Defaucheux C Karabajakian A. Hyperprogression during anti-PD-1/PD-L1 therapy in patients with recurrent and/or metastatic head and neck squamous cell carcinoma. Ann Oncol. 2017;28:1605–1611. 28419181 10.1093/annonc/mdx178

[70] van den Bijgaart RJ Eikelenboom DC Hoogenboom M Futterer JJ den Brok MH Adema GJ. Thermal and mechanical high-intensity focused ultrasound: perspectives on tumor ablation, immune effects and combination strategies. Cancer Immunol Immunother. 2017;66:247–258. 27585790 10.1007/s00262-016-1891-9 PMC5281669

[71] Dirks I Keyaerts M Dirven I Neyns B Vandemeulebroucke J. Development and validation of a predictive model for metastatic melanoma patients treated with pembrolizumab based on automated analysis of whole-body [^18^F]FDG PET/CT imaging and clinical features. Cancers (Basel). 2023;15:4083. 37627111 10.3390/cancers15164083 PMC10452475

[72] Seban RD Moya-Plana A Antonios L. Prognostic ^18^F-FDG PET biomarkers in metastatic mucosal and cutaneous melanoma treated with immune checkpoint inhibitors targeting PD-1 and CTLA-4. Eur J Nucl Med Mol Imaging. 2020;47:2301–2312. 32206839 10.1007/s00259-020-04757-3

[73] Vercellino L de Jong D Dercle L. Translating molecules into imaging: the development of new PET tracers for patients with melanoma. Diagnostics (Basel). 2022;12:1116. 35626272 10.3390/diagnostics12051116 PMC9139963

[74] Wissler HL Ehlerding EB Lyu Z. Site-specific immuno-PET tracer to image PD-L1. Mol Pharm. 2019;16:2028–2036. 30875232 10.1021/acs.molpharmaceut.9b00010 PMC6521698

[75] Robert C Karaszewska B Schachter J. Improved overall survival in melanoma with combined dabrafenib and trametinib. N Engl J Med. 2015;372:30–39. 25399551 10.1056/NEJMoa1412690

[76] Dummer R Ascierto PA Gogas HJ. Encorafenib plus binimetinib versus vemurafenib or encorafenib in patients with BRAF-mutant melanoma (COLUMBUS): a multicentre, open-label, randomised phase 3 trial. Lancet Oncol. 2018;19:603–615. 29573941 10.1016/S1470-2045(18)30142-6

[77] Ascierto PA McArthur GA Dreno B. Cobimetinib combined with vemurafenib in advanced BRAF(V600)-mutant melanoma (coBRIM): updated efficacy results from a randomised, double-blind, phase 3 trial. Lancet Oncol. 2016;17:1248–1260. 27480103 10.1016/S1470-2045(16)30122-X

[78] Guo J Carvajal RD Dummer R. Efficacy and safety of nilotinib in patients with KIT-mutated metastatic or inoperable melanoma: final results from the global, single-arm, phase II TEAM trial. Ann Oncol. 2017;28:1380–1387. 28327988 10.1093/annonc/mdx079 PMC5452069

[79] Robert C Long GV Brady B. Nivolumab in previously untreated melanoma without BRAF mutation. N Engl J Med. 2015;372:320–330. 25399552 10.1056/NEJMoa1412082

[80] Blank CU Lucas MW Scolyer RA. Neoadjuvant nivolumab and ipilimumab in resectable stage III melanoma. N Engl J Med. June 2, 2024 [Epub ahead of print]. 10.1056/NEJMoa2402604 38828984

